# *Leishmania donovani* Induced Cutaneous Leishmaniasis: An Insight into Atypical Clinical Variants in Sri Lanka

**DOI:** 10.1155/2019/4538597

**Published:** 2019-05-27

**Authors:** Yamuna Siriwardana, Bhagya Deepachandi, Chalukya Gunasekara, Wipula Warnasooriya, Nadira D. Karunaweera

**Affiliations:** ^1^Department of Parasitology, Faculty of Medicine, University of Colombo, Colombo 00800, Sri Lanka; ^2^National Hospital, Colombo 00800, Sri Lanka; ^3^Teaching Hospital, Kurunegala 60000, Sri Lanka

## Abstract

Sri Lanka is a recent focus having* Leishmania donovani* induced cutaneous leishmaniasis (CL) as the main clinical entity. A separate clinical entity within profile of CL was described in this study. Laboratory confirmed cases of CL (n= 950, 2002-2014) were analysed. Most lesions showed known classical developmental stages of CL (CCL) observed in other CL endemic settings while few cases (13%, 122/950) showed atypical skin manifestations (ACL). Clinical, geographical, and treatment response patterns of ACL were different from those of CCL. ACL was mainly found among males (68.0%), in 21-40 year age group (51.6%), and reported delayed treatment seeking (23.5% vs 16.3% in CCL), more nonclassical onset (lesions other than acne form <1cm sized papules), (12.1 vs 2.7%, P<0.05.), more head and neck lesions (41.5%. vs 27.2%), more large lesions (>4cm), (18.6 vs 9.9%), and poor laboratory positivity rates (65.6% vs 88.2%) when compared to CCL. When compared to lesions reporting a typical onset, lesions reporting nonclassical onset were more likely to develop ACL later on (50.1% vs 10.7%). As compared to lesions on limbs, those on head and neck and trunk were more likely to be ACL (7.0%, 16.3%, and 22.8%, respectively, P<0.05). ACL features were not age or gender dependent. Highest proportion within ACL category (32.8%) and small proportion of CCL (10.1%) originated from less leishmaniasis prevalent areas (other regions) (P<0.05). North reported more ACL than South (15.9% vs 7.4%). A total of 95 CL cases with a significant travel history were further analyzed. Residents of other regions when acquired infection from North or South developed more ACL than residents in North or South (60.9% vs 15.9% and 42.9% vs 7.4% respectively). Patients in other regions when travelled to North developed more ACL than when they travelled to South (60.9%, 42.9%). ACL and CCL required an average of 18 doses over 16.7 months and 10 doses over 12 weeks, respectively, to achieve a complete clinical cure. Underlying host immunological factors, parasite strain variations and regional variations of both could be underlying etiologies. Established independent transmission within less leishmaniasis prevalent regions combined with an unusual clinical picture leading to poor clinical suspicion and low laboratory confirmation rate will pose potential difficulties in early case detection in these highly populated and commercialized areas. This in turn will further facilitate silent and high disease transmission.

## 1. Introduction

Different clinical entities in leishmaniasis (i.e., cutaneous; CL, visceral; VL and mucosal leishmaniasis; MCL) are mainly determined by the infecting species that are multiple among the genus* Leishmania*. CL is the commonest reported clinical form affecting many tropical and sub-tropical countries with an increasing incidence [1, 2]. Classical leishmanial ulcer develops over an exposed body area from a painless acne-form papule that enlarges and eventually ulcerate at its summit. Lesions often remain single and are known to self-heal eventually.

Visceralizing* Leishmania* affect internal organs and clinically apparent untreated VL is fatal.

VL is known to cause great mortality and morbidity [3–5]. Despite regional variation there is significant decrease in the number of new VL cases in South east Asian region and also some decrease in other parts of the world that has led WHO to revision of the global estimates [3, 6–13], in spite of the regional drive that aims at disease elimination in Indian subcontinent by year 2020.

A genetic variant of* L. donovani, *the usual cause of VL, has been reported to cause a large outbreak of CL in Sri Lanka since year 2001 [14–16]. Widening dimensions of CL outbreak [17–19], micro-changes within the continued profile of CL [20], varied risk factors for transmission [21], case clustering of CL in communities [22], poor treatment response [23], CL associated humoral response [24] and emergence of few MCL and VL cases [25–27], and species/strain variation [28] are of recent local concern. Urgent need for disease control has been highlighted at several occasions [29, 30].* L. donovani *is mainly anthroponotic in the Asian region, and reduction of human infection is considered critical in* L. donovani* control [1, 31]. However, nondisturbing nature of most initial skin lesions leads to poor self-referral. Absence of pathognomonic clinical features in leishmaniasis often makes the clinical diagnosis difficult. CL caused by* L donovani *is still rare and literature on this clinical entity is scarce [32–34]. Previous studies in Sri Lanka identified useful clinical markers for field screening and case interpretation in classical CL (CCL) [35]. Atypical manifestations of CL (ACL) were first identified in a minority during the early stages of the epidemic [18].

ACL comprise an important category that can complicate initial clinical screening leading to late or misdiagnosis of CL. Many clinical forms including erysepeloid, zosteriform, disseminated, lupoid, and verrucous lesions have been described in association with classical CL [36–39]. Genetic variation, altered immune pathological sequences, and varied treatment response have also been identified or suggested as underlying reasons for ACL [40, 41]. Understanding the early and late presentations, immediate and long-term sequelae, outcome of treated and untreated lesions, and aetiology of different clinical variants is important for patient management and disease containment. Current study aimed at describing the atypical clinical forms associated with* L. donovani *induced CL in Sri Lanka.

## 2. Materials and Methods

Patients investigated at the Center for research, training, and diagnosis of leishmaniasis, in University of Colombo (late 2002–early 2014), with suspected skin lesions of cutaneous leishmaniasis were recruited after informed consent. Clinical evaluation and sample collection were carried out by PI/ qualified medical officers and trained technical officers, respectively. Laboratory confirmation was established by microscopy, culture or PCR [42, 43]. Demographic, travel, and treatment data of laboratory positive cases and clinical data pertaining to a randomly selected single lesion (in single or multiple lesions) of each patient were collected. An equal number of time matched (presented within a month) cases of CCL were considered together with all laboratory confirmed cases of ACL for analysis of travel data and geographical variations. A subset of 40 patients with laboratory confirmed ACL were followed up for a period of 2 years to analyse treatment outcome. Missing and doubtful information and case histories with inadequate information were excluded on a case by case basis or lesion wise. Data analysis was carried out using SPSS.

### 2.1. Working Definitions

#### 2.1.1. Classical Primary Lesion

Single skin papule of ≤1cm diameter.

#### 2.1.2. Classical Lesion Stages

**Figure 1:**
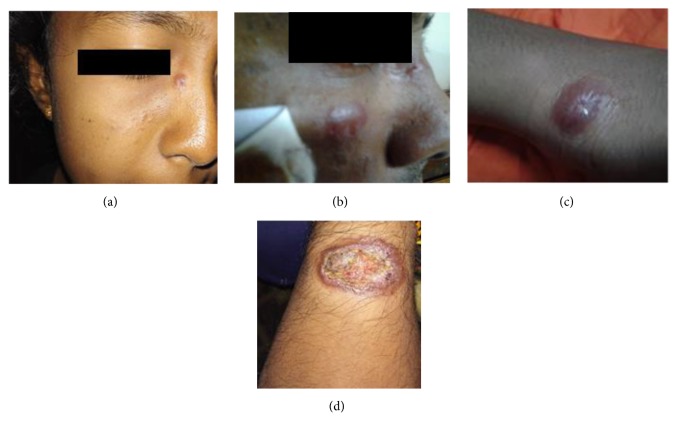
Typical developmental stages of a leishmanial skin lesion observed in the study population. (a) papular lesion on the face, (b) enlarging nodular lesion on the face, (c) early ulcerating nodule on the forearm, and (d) completed ulcer on the forearm.

Main CL picture of* L.donovani* induced CL in Sri Lanka is known to be similar to the same observed with regard to the traditionally CL causing species of* Leishmania *in other parts of the world. Therefore, known developmental stage of a leishmanial skin lesion; nodule, ulcerating nodule, or completed ulcer was considered as classical stages (Figure 1).

#### 2.1.3. Atypical Lesion Stages

Any other form of laboratory confirmed skin lesions of CL other than CCL observed in the study population.

#### 2.1.4. Geographical Regions (Figure 2)

Northern region: area covering 6 districts in North.

Southern region: area covering 5 districts in South.

Other regions: Central, Western and Eastern regions covering 7, 4 and 3 districts respectively.

#### 2.1.5. Significant Travel History

Travel to areas other than patient's resident district is within preceding 24 months.

### 2.2. Ethical Aspects

Ethical clearance was obtained from the Ethics Review Committee of Faculty of Medicine, University of Colombo, Sri Lanka.

## 3. Results

A total of 1125 clinically suspected lesions (including 186 lesions clinically diagnosed as ACL) were considered for the analysis. Light microscopy, IVC, or PCR methods confirmed CL in 950/1125 patients. There were 12.8% (122/950) ACL cases and 87.2% (n=828/950) CCL among the laboratory confirmed patients. Laboratory confirmed group was considered for further analysis. Parasite positive rate of ACL was less as compared to that of CCL (65.6%, 122/186 vs 88.2% (n=828/939). It did not vary significantly over the course of ACL, except for a slight reduction observed in chronic lesions (60% in >1 year lesions), (data not tabulated).

Majority of patients with atypical lesions were between 21 and 40 years of age (n=63/122, 51.6%). Males were affected more than females (83/122, 68.0%). There were no gender associated differences between proportions of CCL and ACL in total population (Table 1). However proportion of 21-40 year old cases with ACL increased significantly with lesion duration (Tables 1 and 2).

### 3.1. Clinical Profiling

ACL were nonulcerative lesions (76.2%), generalized rashes (12.3%) or other forms. Most remained as single lesions (101/122, 82.8%). There were 13.8% and 17.2% multiple lesions in CCL and ACL groups, respectively. These proportions remained almost similar at early and late durations of lesion within the course of an ACL lesion (18.2 and 14.8%) (Table 3). Higher proportion of ACL as compared to CCL had presented lately for leishmaniasis investigations (23.5% vs 16.3%). However, nearly a half of ACL lesions presented within 4 months of duration of lesion (55/115, 47.8%). Both CCL and ACL mainly reported a typical onset with a classical primary lesion (97.3% and 81.9%, respectively). Only a few lesions reported other features at the onset (multiple acne-form papules, macular rash, discolored patchy, etc.). However, proportion of ACL started as nonclassical primary lesions was significantly higher as compared to that of CCL (12.1% vs 2.7%) (Table 2). In this group, 15/31, 50% developed into atypical stages subsequently also. Almost all the lesions starting as a classical primary lesion developed in to chronic CCL later on 89.3% while only 50% of lesions starting as other types developed in to CCL (Table 2). However long-standing ACL lesions reported a typical onset as compared to early ACL lesions (Table 2). More than a half of ACL lesions remained small (<2cm) (61/113, 54%) while 18.6% of lesions were large (>4cm). Proportion of large ACL lesions were higher as compared to that of CCL (18.6% vs 9.9%), (Table 1). ACL occurred more on head and neck area (41.5%) followed by distal parts of limbs (29.2% in forearms and legs). However ACL lesions presented mainly on head and neck region while CCL lesions were on distal limbs (Table 2). Truncal lesions were more likely to be atypical lesions as compared to lesions developed on other sites (22.8%, 16/70), (Table 1). Higher proportion of ACL lesions was irregular in shape as compared to classical lesion stages (41.2% vs 8.5%). Nearly 40% of ACL became irregular in shape at an early stage and this proportion did not increase over time (Table 3).

### 3.2. Progress of Lesions

Multiplication and enlargement was shown in both early and late categories (Table 3). In early ACL lesions 53% were small (<2cm), while 20 % were large (>4cm). In chronic atypical lesions nearly equal proportions of both small and larger lesions were seen (Table 3).

Lesions became more irregular in shape with increasing lesion duration (Table 3).

### 3.3. Geographical Distribution, Resident, and Visiting Populations (Table 4 and Figure 3)

Even though CCL was mainly observed in all the regions, geographical distribution pattern of ACL was significantly different from that of CCL (Table 4). More than a third of lesions reported in less prevalent leishmanial areas (other regions) were ACL while North and South has lesser proportions of ACL (P<0.05), (Table 4). North reported more ACL than South (Table 4).

Out of the total group of 244 (122 ACL cases and time matched 122 CCL cases), 95/244 (38.9%) reported a significant travel history. Nearly a half of them originated from other regions (49/95, 51.5%). Patients revealed a travel history in North (23/49), South (7/49), or the same region (19/49). Patients travelled in North or within the same region developed more ACL than those who travelled in South (60.9% and 57.9% vs 42.9%) (Table 4). Proportion of ACL reported in those who travelled from other regions to North was clearly higher than proportion of ACL in residents of North (60.9% vs 15.9% in Table 4). Similarly, proportion of ACL in patients travelled from other regions to South was also higher than that of residents in South (42.9% vs 7.4% in Table 4). Rest of the group travelled only in areas within the same resident region also reported higher proportions of ACL as compared to that of total patient population in the region (57.9%, 32.8% in Table 4).

Rest of the group with a significant travel history (46/95) originated from either Northern or Southern regions in Sri Lanka. However, they had ACL lesion proportions consistent with the ACL proportions observed for the resident region, irrespective of their travel area (travel data not shown).

### 3.4. Treatment Response

An average of 18 doses of weekly or daily SSG and 16.7 months were taken by the patients with ACL to achieve a complete clinical cure of the lesions while average 10 doses over 12 weeks required for a CCL to complete clinical cure as evidenced by epithelialization. Three ACL lesions were on treatment at the time of analysis and remained inconclusive.

### 3.5. Different Presentations in Some Patients (Figure 4)

Detailed clinical observations of some individual cases of atypical CL are given below. An adult male (41 years) from Western region reported an irregular plaque that started with an atypical onset (a rash) on the earlobe. During the first 18 months the lesion developed in to a hyper-pigmented irregular plaque of approximately 5 cm in largest diameter. Surface scaling of lesion was observed. There was no sporotrichoid spread or clinical features suggestive of visceralization. Patient did not report a travel history to other regions

He was in the same region during the preceding two years, in Central region, prior to that and had annual visits to Northern region for past 8 years. He was treated with 42 doses of IM-SSG, 13 doses of IL-SSG, and 2 doses of cryotherapy for 8 months. The lesion poorly responded to treatment and after 22 months of posttreatment, an exacerbation of the lesion was seen. Patient is still under treatment.

**Figure 4:**
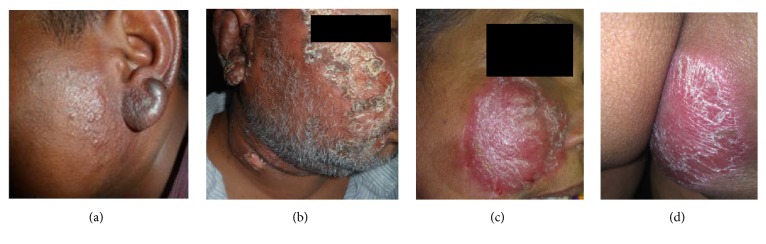
Atypical CL lesions observed in the study population. (a) Case 1, (b) case 2, (c) case 3-lesion I, and (d) case 3-lesion II.

An elderly male from Southern region presented with irregular erythematous ulcerative patches on head and neck region (Figure 4(b)). Lesion started as a macule of approximately 5 cm in diameter. The ulcer showed well-demarcated sharp edge with regular base and dried crust. There were no surrounding skin changes or clinical evidence for visceral disease. Lesions were cured with 40 doses of IM-SSG and 17 doses of cryotherapy within 15 months.

A resident female, from Western region, presented with two rounded, atypical plaque type lesions in right face and buttock of 1 month and 2 weeks duration respectively (Figures 4(c) and 4(d)). She worked in Batticaloa in Eastern region 8 months prior to lesion onset. Patient had a typical classical primary lesion which enlarged acutely to a size of approximate 8 cm in diameter. Lesions were erythematous and had well-defined margins with irregular edges. Both lesions had scaly surfaces. Surrounding skins showed inflammation, scaling, and no satellite lesions or sporotrichoid spread. On examination, she had no symptoms associated with visceral disease. A clinical cure was achieved with 6 intralesional doses of sodium stibo gluconate.

## 4. Discussion

Clear majority of skin infections resulted in CCL over the past several years in this setting [14, 17, 19–21]. Meanwhile approximately a tenth of skin infections developed into ACL and further showed deviations from CCL. Most clinically straight forward lesions are either treated on clinical grounds or investigated in the hospital setting with the University center's establishment of microscopy training for government technical staff in health care institutions. An increasing trend to refer only nonclassical lesions to the specialized central laboratory was observed as a result during the latter part of the epidemic. Therefore, observed ACL prevalence is likely to be a slight exaggeration of the true figure, though these numbers were minimal and did not probably altered the overall figures considerably. ACL is of concern when changing trends within the CCL profile, poor treatment response, growing numbers of CL, emergence of VL, and presence of a genetic variant of a dangerous parasite are considered.

Age and gender association in ACL were consistent with previous findings for CCL in the country [20]. Zosteriform, sporotrichoid, acne-form generalized, erysepeloid, or plaque type lesions were observed within the ACL group.

Even though most ACL lesions started a classical primary lesion some reported a range of non-classical initial lesions including generalized body rashes, multiple acne-form papules, or hypopigmented macular lesions. These lesions are likely to be misdiagnosed at the onset. Non classical onset also indicated high possibility for development of ACL later on. Atypical lesions were shown to develop both during early and late stages of the course of a lesion and associated with a poor laboratory confirmation rate. This indicates the low clinical screening power leading to referral of nonleishmanial cases, low parasitaemias, or both. Broad clinical picture of ACL was also different from that of CCL. Most remained nonulcerative and irregular in shape. Lesions that occur on back, trunk, head, and neck areas may have a tendency to develop in to ACL, a phenomenon observed in other settings as well [40]. Accurate detection of these is important as the general accepted norm is to suspect CL in rounded or oval shaped single lesions developing from classical primary lesions over exposed and peripheral body areas. ACL lesions tend to run a chronic course and/or self-referred later on to clinical setting as compared to CCL.

FGT is a crude test used to exclude VL. Parasitological assays used in leishmaniasis confirm infection in a given site while serology provides indirect evidence. But complete differentiation of VL from CL is difficult. Combination of clinical and laboratory results is usually considered in arriving at a diagnosis of either VL or CL. Skin lesion with evidence for parasites in the absence of systemic clinical features are treated as CL. Cases with systemic clinical features with parasite positivity in suitable internal tissue or indirect serological evidence are treated as VL. There can be a theoretical possibility of ACL in Sri Lanka to visceralize concurrently or subsequently, though this needs in depth studies to arrive at a conclusion. However, previous clinical studies have failed to demonstrate Visceralization of CL in this focus.

A proportion of ACL lesions remained small and single while the rest multiplied and enlarged as irregular shaped lesions during early or late stages during lesion development.

Local CL infection could be assumed to be confined to skin as previously reported [44]. Diagnostic clinical markers were previously developed for CL based on this assumption [35]. Presence of enlarged, irregular, and multiplied atypical lesions in some patients in both early and late durations may indicate possible individual variation in ACL progression.

At present, two main disease reporting areas are observed in Southern and Northern regions in the country [20]. But both CCL and ACL lesions were reported from diverse localities within the island. However, proportion of ACL occurring in other regions is clearly higher as compared to that of North and South. Northern focus seems to report more ACL as compared to South probably indicating a geographical variation of circulating parasite strains. Also proportion of ACL in resident CL patients of other regions travelled to North and South seems to be higher as compared to that of resident CL patients from North or South who probably acquired the infection within the resident area though they had a significant travel history to other districts or regions.

It may be assumed that the residents in other regions are nonimmune or less immune to leishmanial infections. Such individuals acquiring the infection while travelling in disease prevalent areas may be more likely to develop atypical lesions than the patients acquiring infections in immune populations. This may indicate a role of host immune mechanisms in clinical outcome. Final observed outcome may be the result of a possible combined role of the nonimmune host developing more ACL and geographical variations in circulating parasite strains with a slight influence from the different case referral patterns practiced by the clinicians. Regional variation in epidemiological characteristics is already known in this setting [21, 22]. Though it is not the focus of this publication, this may be worth the attention. Final clinical outcome, long-term sequalae, drug response patterns, and therefore the desired disease control methods may also be different in these two areas within the country.

Study also revealed that a considerable proportion of cases residing in other regions acquire leishmaniasis without a travel history to North or South. Circulating parasite variants in less leishmaniasis prevalent areas may result in establishment of infection in these areas further complicating clinical judgments and disease control.

Atypical lesions seem to demonstrate delayed response to the first line antileishmanial treatment as shown in some other studies as well [40, 45]. This provides further supportive evidence for the possible strain differences between the* Leishmania *sp. causing ACL or CCL in this endemic setting. Geographical location of the patient, likely place of acquiring infection, nature of primary lesion, and affected body site, seems to play a role in eventual development of a lesion in to ACL.

## 5. Conclusions

Current analysis demonstrated that dermotropic variants of local L. donovani infecting humans results in a variety of atypical lesions in a minority of infections and detailed the clinical profile and treatment outcome of this clinical entity for the first time. Underlying host or parasitic etiologies and ACL associated poor treatment response can further complicate the situation. This clinical entity thus deserves careful attention of the clinician, epidemiologist, and the researcher. Curative sector needs measures to encourage early referrals, better professional awareness, high degree of clinical suspicion, and adopting more sensitive diagnostic tools before arriving at a negative conclusion especially in less leishmanial prevalent areas [41]. Recently developed modified single tube nested PCR method may be used for disease confirmation in microscopically negative ACL [45]. Unknown sequalae associated with ACL can further complicate disease combatting efforts in an endemic setting. Parasite strain association and a role of host factors or both could be underlying etiologies of which understanding will facilitate appropriate handling of this clinical entity. Study of demographics, clinic, immunological, and parasitological factors in Northern and Southern leishmaniasis transmission foci are indicated.

## Figures and Tables

**Figure 2:**
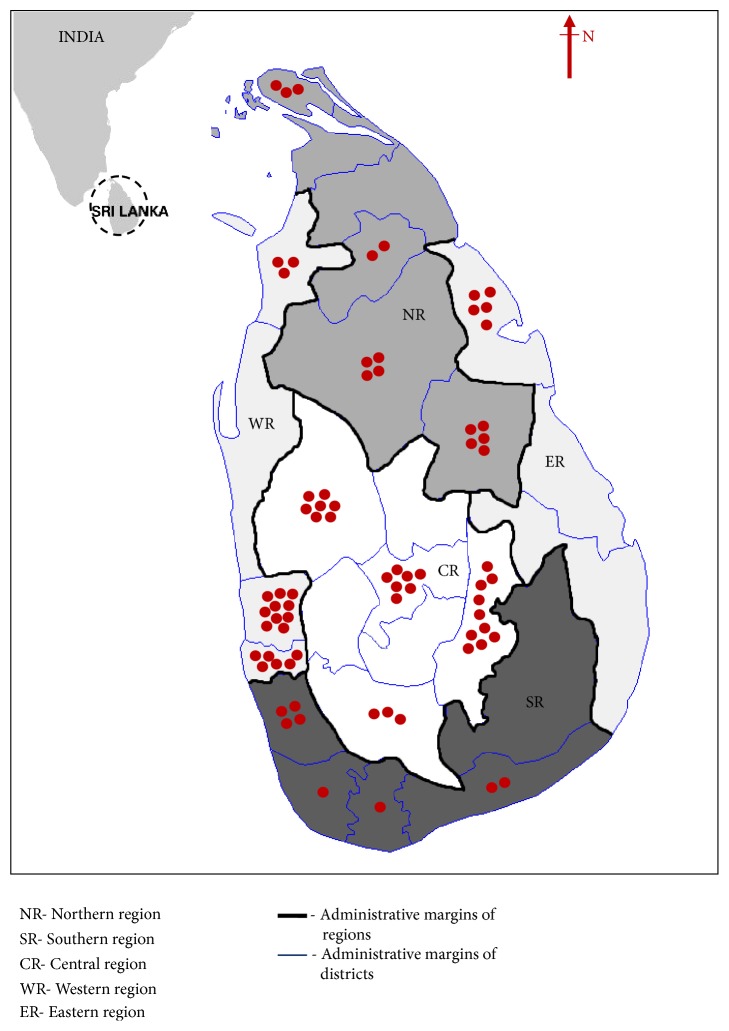
Distribution of atypical cases of CL within the five different geographical regions within the country. Percentages of ACL cases were calculated based on total cases in each district. One dot represents 5%. Dot counts were calculated to the nearest total multiplications of 5.

**Figure 3:**
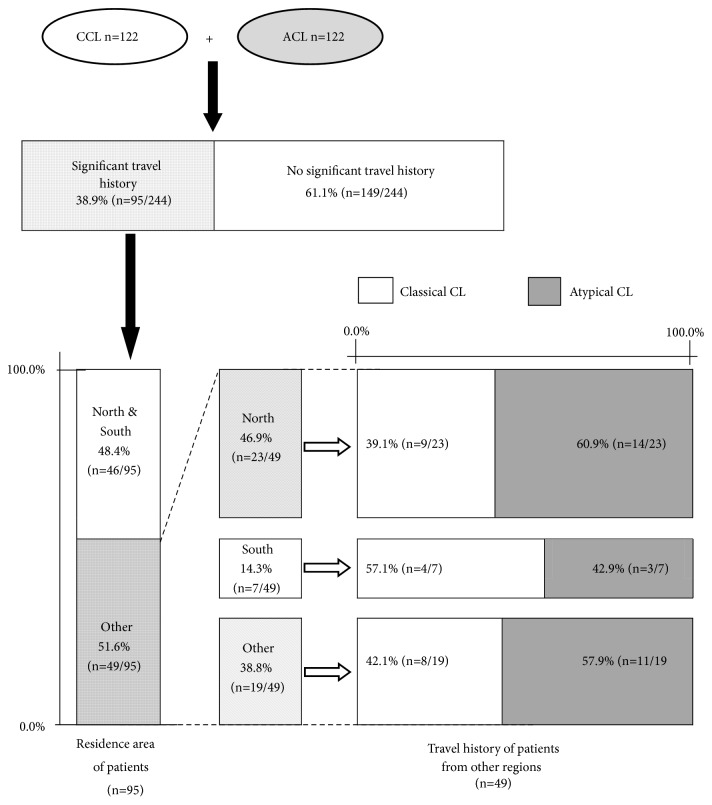
Association of CCL and ACL with travel history in subgroup (n=95) with a significant travel history in the study population.

**Table 1:** Sociodemographic features associated with atypical lesions.

Feature	Typical		Atypical		P value
	Count	percentage	Count	percentage	
*Age (years)*					
<20	191	23.1	19	15.6	P>0.05
21-40	381	46.0	63	51.6	
>40	256	30.9	40	32.8	
Total	828	100.0	122	100.0	

*Gender *					
Male	555	67.0	83	68.0	P>0.05
Female	273	33.0	39	32.0	
Total	828	100.0	122	100.0	

**Table 2:** Clinical characteristics of typical and atypical lesions in the study population.

	Classical lesions		Atypical lesions		P value
*Lesion duration (months)* Mean (SD)	6.8 (9.53)		10 (26.47)		

*Feature∗*	Count	Percentage	Count	Percentage	

*Number of lesions*					
Single	714	86.2	101	82.8	
Multiple	114	13.8	21	17.2	
Total	828	100.0	122	100.0	

*Lesion duration∗ (months)*					
Up to 4	411	49.6	55	47.8	
5-10	282	34.1	33	28.7	
Over 10	135	16.3	27	23.5	
Total	828	100.0	115	100.0	

*Onset∗*					
Typical	567	97.3	68	81.9	P<0.05
Other	16	2.7	15	12.1	
Total	583	100.0	83	100.0	

*Size of lesion∗ (cm)*					
Up to 2cm	540	65.2	61	54.0	P<0.05
2-4 cm	206	24.9	31	27.4	
Above 4cm	82	9.9	21	18.6	
Total	828	100.0	113	100.0	

*Site of lesion∗*					
Limbs	549	66.3	46	43.4	P<0.05
Trunk	54	6.5	16	15.1	
Head and neck	225	27.2	44	41.5	
Total	828	100.0	106	100.0	

*Shape of lesion∗*					
Round or oval	738	91.5	67	58.8	P<0.05
Irregular	69	8.5	47	41.2	
Total	808	100.0	114	100.0	

*∗*Missing and doubtful information was excluded lesion wise.

**Table 3:** Sociodemographic and clinical trends during development of ACL.

Feature*∗*	Early ACL (<4months)		Chronic ACL (>10months)		P value
	Count	Percentage	Count	percentage	
*Sex*					
Male	34	61.8	21	77.8	
Female	21	38.2	6	22.2	
Total	55	100.0	27	100.0	

*Age *					
1.00	11	20.0	4	14.8	P<0.05
2.00	25	45.5	18	66.7	
3.00	19	34.5	5	18.5	
Total	55	100.0	27	100.0	

*Number *					
Single	45	81.8	23	85.2	
Multiple	10	18.2	4	14.8	
Total	55	100.0	27	100.0	

*Onset *					
As primary lesion	29	72.5	18	90.0	
Other types	11	27.5	2	10.0	
	40	100.0	20	100.0	

*Size of lesions*					
<2cm	27	52.9	14	58.3	
2-4cm	14	27.5	5	20.8	
>4cm	10	19.6	5	20.8	
Total	51	100.0	24	100.0	

*Shape of lesion*					
Round or oval	33	62.3	14	53.8	
Irregular	20	37.7	12	46.2	
Total	53	100.0	26	100.0	

*∗*all missing or doubtful information was excluded lesion wise.

**Table 4:** Distribution of CCL and ACL in patients from different regions within the country.

Feature	CCL		ACL		Total		P value
	Count	Percentage	Count	Percentage	Count	Percentage	
*Residential areas of patients*							
North	196	84.1	37	15.9	233	100.0	P<0.05
South	548	92.6	44	7.4	592	100.0	
Other	84	67.2	41	32.8	125	100.0	

*Travel history of patients in other regions*							
To north	9	39.1	14	60.9	23	100.0	
To south	4	57.1	3	42.9	7	100.0	
Within same region	8	42.1	11	57.9	19	100.0	

## Data Availability

Data is not made available as it was not part of the ethical clearence agreement and due to patient confidentiality.
